# Intranasal Adipose-Derived MSC Extracellular Vesicles Confer Sustained Cognitive Improvement and Suppress Alzheimer’s Pathology in APP/PS1 Mice

**DOI:** 10.3390/biom16060798

**Published:** 2026-05-28

**Authors:** Mengsi Tian, Renjun Feng, Chunmei Gong, Xinyu Ben, Zhijian Ma, Xinan Yi, Qingyun Guo

**Affiliations:** Key Laboratory of Tropical Translational Medicine of Ministry of Education & Key Laboratory of Brain Science Research and Transformation in Tropical Environment of Hainan Province, School of Basic Medical Sciences, Hainan Academy of Medical Sciences, Hainan Medical University, Haikou 571199, China; tianmengsi@muhn.edu.cn (M.T.); hy0206183@muhn.edu.cn (R.F.); hy0206234@muhn.edu.cn (C.G.); benxinyu@muhn.edu.cn (X.B.); hy0108003@muhn.edu.cn (Z.M.)

**Keywords:** Alzheimer’s disease, adipose-derived mesenchymal stem cells, extracellular vesicles, intranasal delivery, cognitive impairment, amyloid pathology

## Abstract

Alzheimer’s disease (AD) lacks effective disease-modifying therapies, and extracellular vesicles (EVs) derived from adipose-derived mesenchymal stromal cells (ADMSCs) have emerged as promising therapeutic candidates. In this study, we investigated the brain biodistribution and dose-dependent effects of intranasally administered ADMSC-EVs in female APP/PS1 mice, with age-matched wild-type mice and vehicle-treated transgenic mice serving as controls. EV biodistribution was assessed using PKH26 labeling, cognitive performance was evaluated using the Morris water maze, Y-maze, and novel object recognition tests, and hippocampal amyloid pathology and plasma AD-related biomarkers were analyzed. Intranasally delivered ADMSC-EVs rapidly reached multiple brain regions, including the hippocampus, improved learning and memory performance, and reduced hippocampal amyloid-β 1-42 (Aβ42) deposition and plaque burden. These effects followed a nonlinear dose–response pattern, with reduced efficacy at low doses and no additional benefits at high doses. Notably, partial behavioral and pathological benefits persisted after treatment cessation. Together, these findings show that intranasal ADMSC-EVs exert therapeutic effects in APP/PS1 mice and support the importance of dose optimization and post-treatment durability in the development of EV-based interventions for AD.

## 1. Introduction

Alzheimer’s disease (AD) is a progressive neurodegenerative disorder and the leading cause of dementia, accounting for 60–80% of all cases worldwide. It represents a major contributor to disability and mortality in the aging population [1,2], affecting approximately 50 million people globally, with the number projected to exceed 150 million by 2050 [3]. Although key pathological features, including amyloid-β (Aβ) accumulation, tau pathology, cholinergic dysfunction, and neuroinflammation, have been extensively characterized, the mechanisms driving disease progression remain incompletely understood [4,5]. Currently, therapies provide only limited symptomatic benefits without preventing neurodegeneration.

Mesenchymal stem cells (MSCs) have emerged as a promising therapeutic strategy for neurodegenerative diseases [6,7]. Growing evidence suggests that their beneficial effects are largely mediated by extracellular vesicles (EVs), nanosized particles enriched in bioactive nucleic acids, proteins, and lipids [8,9]. Among MSC sources, adipose-derived MSCs (ADMSCs) are particularly attractive due to their accessibility and favorable safety profile. ADMSC-derived EVs retain the immunomodulatory and neuroprotective properties of MSCs while avoiding the risks associated with cell transplantation [10,11]. Previous studies have shown that MSC-EVs modulate inflammatory responses [12,13,14], protect neurons and synapses [15,16], promote axonal growth [17,18], reduce Aβ deposition, and improve cognitive deficits in several AD models, including human amyloid precursor protein/presenilin-1 (APP/PS1) and triple-transgenic Alzheimer’s disease (3xTg-AD) mice [19,20,21].

EVs transfer diverse nucleic acids, including messenger RNA (mRNA), microRNA (miRNA), long noncoding RNA (lncRNA), and DNA, to recipient cells. Among these, miRNAs are key regulators of cell growth, differentiation, angiogenesis, and apoptosis [22,23,24]. ADMSC-EVs are enriched in multiple neuroprotective miRNAs, such as miRNA-21, miRNA-143, and miRNA-222 [25]. In particular, miRNA-21 has been shown to attenuate Aβ-induced neuronal apoptosis via the programmed cell death 4/phosphoinositide 3-kinase/protein kinase B/glycogen syn-thase kinase-3 beta (PDCD4/PI3K/AKT/GSK-3β) pathway [25] and is increasingly recognized as a potential biomarker and therapeutic target for central nervous system (CNS) disorders [6,26]. These findings support the concept that ADMSC-EVs may function as natural carriers that deliver functional miRNAs across the blood–brain barrier (BBB) to modulate AD-related pathology [27].

Although the therapeutic potential of MSC-derived EVs in AD models has been increasingly reported, several translationally important issues remain insufficiently defined. In particular, the optimal intranasal dosing window, the nonlinear dose–response characteristics, and the durability of therapeutic benefits after treatment discontinuation have not been systematically evaluated within a single experimental framework. Here, we investigated the dose-dependent efficacy and brain biodistribution of intranasal ADMSC-EVs in APP/PS1 mice. Behavioral performance, amyloid pathology, and transcriptomic changes were assessed, and the persistence of therapeutic benefits after treatment withdrawal was evaluated. Our findings reveal a nonlinear dose–response relationship, highlighting the importance of dose optimization for future EV-based therapies in AD.

## 2. Materials and Methods

### 2.1. Extraction, Culture, and Characterization of ADMSCs

Human adipose tissue was obtained under sterile conditions from a healthy female donor undergoing elective liposuction. The protocol was approved by the Ethics Committee of Hainan Medical University (Approval No. HYLL-2023-026), and written informed consent was obtained. Tissue was processed for cell isolation as previously described [28]. After natural stratification, the intermediate fatty layer was collected and digested with type I collagenase in phosphate-buffered saline (PBS) until a uniform suspension was achieved. The digest was centrifuged, washed, filtered, and resuspended in complete culture medium. Nonadherent cells were removed after 24 h, and adherent cells were expanded and used at passages 2~5. Multilineage differentiation was assessed to confirm MSC identity. For adipogenic, chondrogenic, and osteogenic differentiation, ADMSCs were cultured in lineage-specific induction media for 14~21 days and stained with Oil Red O, Alcian Blue, or Alizarin Red, respectively. Immunophenotyping was performed on passage-3 ADMSCs using fluorochrome-conjugated antibodies against CD105 and CD90 (positive markers) and CD19 and CD11b (negative markers), followed by flow cytometry. EV marker expression (CD63, TSG101, ALIX) was confirmed by Western blotting.

### 2.2. Isolation and Identification of ADMSC-Derived EVs

EVs were isolated from the serum-free conditioned medium collected 72 h after ADMSC culture. Sequential centrifugation (300× *g*, 2000× *g*, and 10,000× *g* for 10 min each, 4 °C) removed cells and debris. The supernatant was filtered through a 0.22 μm membrane, concentrated using ultrafiltration at 5000× *g* for 30 min, and ultracentrifuged at 100,000× *g* for 70 min at 4 °C. The EV pellet was resuspended in sterile PBS. Particle size distribution and concentration were analyzed by nanoparticle tracking analysis (NTA) using a Zetaview system (Particle Metrix, Inning am Ammersee, Germany). EV morphology was evaluated by transmission electron microscopy (TEM). Briefly, the EV-containing supernatant was diluted 1:10,000 in PBS. A 10 μL aliquot of the diluted EV suspension was placed on a Formvar-coated copper grid (Electron Microscopy Sciences, Hatfield, PA, USA) and incubated for 10 min at room temperature. Excess liquid was removed using filter paper, and the grid was negatively stained with 1% phosphotungstic acid solution (79690, Sigma, Shanghai, China) for 70 s. After removing the excess staining solution, the grid was dried for 5 min. Images were acquired using a transmission electron microscope (Hitachi HT-7800, Tokyo, Japan) at an accelerating voltage of 60 kV.

### 2.3. PKH26 Labeling and In Vivo Brain Distribution of EVs

EVs were labeled with PKH26 fluorescent dye according to the manufacturer’s instructions. EV pellets were resuspended in Diluent C, mixed with an equal volume of PKH26 solution (5 μL PKH26 in 0.5 mL Diluent C), and incubated for 5 min, and staining was terminated with serum-free medium. Labeled EVs were ultracentrifuged at 100,000× *g* for 70 min, washed twice to remove residual dye, and resuspended in PBS. For in vivo tracing, PKH26-labeled EVs were administered intranasally to the mice once daily for three days. One hour after the final administration, brains were collected for cryosectioning and fluorescence microscopy. A dye-only control was processed in parallel to exclude signals from the free PKH26.

### 2.4. Animals and EV Administration

Seven-month-old female APP/PS1 mice and age-matched C57BL/6J wild-type (WT) mice (20~25 g) were obtained from the Guangdong Medical Laboratory Animal Center (Guangzhou, China). All procedures were approved by the Animal Experiment Ethics Committee of Hainan Medical University (Approval No. HYLL-2023-026) and conducted in accordance with the institutional and ARRIVE 2.0 guidelines. Mice were housed under standard conditions (12 h light/dark cycle, 22 ± 2 °C, 50 ± 5% humidity) with ad libitum access to food and water. For the 8-week treatment study, mice were randomly assigned to six groups (*n* = 8 per group): WT control, APP/PS1 control, three EV-treated APP/PS1 groups (5 × 10^7^, 2 × 10^8^, or 2 × 10^9^ particles per 10 μL), and an oxiracetam-treated APP/PS1 group (100 mg/kg, intraperitoneally). EVs or PBS were administered intranasally five times per week for 8 weeks. For the treatment withdrawal experiment, a separate cohort (*n* = 7 per group) received the same 8-week dosing regimen, followed by an additional 8-week treatment-free period to evaluate the durability of therapeutic effects. After behavioral testing, mice were anesthetized with isoflurane (R510-22-10, RWD Life Science, Shenzhen, China; 3% for induction and 1.5% for maintenance; minimum alveolar concentration [MAC] in mice, approximately 1.4%) and then perfused or euthanized for brain collection.

### 2.5. Y-Maze Test

The Y-maze consisted of three identical arms (30 × 8 × 15 cm) positioned 120° apart. Mice were placed in the center and allowed to explore for 8 min. An arm entry was defined as all four paws entering an arm. Spontaneous alternation was defined as consecutive entries into three different arms (e.g., ABC, BCA) as previously described [29]. Spatial working memory was quantified as the percentage of spontaneous alternation: [number of alternations/(total arm entries − 2)] × 100%. The apparatus was cleaned with 70% ethanol between trials.

### 2.6. Novel Object Recognition Test

The novel object recognition test was conducted in an open-field arena (46 × 30 × 16 cm). Mice underwent a 10 min training phase with two identical objects, followed 6 h later by a 5 min testing phase in which one familiar object was replaced with a novel object. Exploration was defined as nose-directed investigation within 1 cm of the object, including sniffing or touching. Recognition memory was assessed by comparing the exploration times of familiar and novel objects.

### 2.7. Morris Water Maze Test

Spatial learning and memory were evaluated using the Morris water maze test. The apparatus consisted of a circular pool (120 cm diameter) filled with water maintained at 22–24 °C. A circular escape platform (10 cm diameter) was placed in the target quadrant. On day 1, visible-platform training was conducted with the platform above the water surface. From days 2–5, hidden-platform training was performed with the platform submerged. Each mouse completed four trials per day from pseudorandomized starting positions. If the platform was not located within 60 s, mice were guided to it and allowed to remain for 10 s. On day 6, a probe trial was performed without the platform. Mice swam freely for 60 s, and memory retention was evaluated by the number of platform-location crossings and the percentage of time and distance spent in the target quadrant.

### 2.8. Tissue Immunofluorescence

Immunofluorescence was performed as described previously [30]. Mice were anesthetized and transcardially perfused with saline, followed by 4% paraformaldehyde (PFA). Brains were post-fixed in 4% PFA and cryoprotected in graded sucrose (10%, 20%, and 30%) at 4 °C. Tissues were coronally sectioned at 20 µm using a cryostat (Leica Microsystems, Wetzlar, Germany). Sections were permeabilized with 0.1% Triton X-100 and blocked in 1% bovine serum albumin (BSA) containing 0.3% Triton X-100 for 30 min. Primary antibodies against glial fibrillary acidic protein (GFAP; 1:2000, Millipore, Billerica, MA, USA), ionized calcium-binding adapter molecule 1 (IbA1; 1:1000, Abcam, Cambridge, MA, USA), neuronal nuclei (NeuN; 1:2000, Abcam), and Aβ42 (1:1000, Proteintech, Wuhan, China) were applied overnight, followed by species-appropriate fluorescent secondary antibodies (1:500, Abcam) for 1 h. Nuclei were counterstained with 4′,6-diamidino-2-phenylindole (DAPI), and sections were mounted with antifade medium. For the analysis of PKH26-labeled EV distribution, adjacent sections were processed in parallel without primary antibody incubation. Images were acquired under identical exposure settings using a fluorescence microscope.

### 2.9. Congo Red Staining

Frozen brain sections were fixed in 4% PFA for 10 min, rinsed with PBS, and stained with Congo red solution for 20 min at room temperature. Sections were differentiated in ammonia water, rinsed, and mounted with antifade medium. Amyloid deposition was visualized using fluorescence microscopy.

### 2.10. Western Blot Analysis

Western blotting was performed as described previously [31]. Cells and EVs were homogenized in radioimmunoprecipitation assay (RIPA) buffer containing phenylmethylsulfonyl fluoride (PMSF), and the protein concentrations were determined using a bicinchoninic acid (BCA) assay kit (BL521A, Biosharp, Shanghai, China). Equal amounts of protein were separated by sodium dodecyl sulfate–polyacrylamide gel electrophoresis (SDS–PAGE) and transferred onto nitrocellulose membranes. Membranes were blocked with 5% BSA in Tris-buffered saline with Tween-20 (TBST) and incubated overnight at 4 °C with primary antibodies, followed by incubation with secondary antibodies for 1 h at room temperature. Bands were visualized using a TANON 5200 Multi imaging system. Primary antibodies were as follows: anti-CD63 (Servicebio, Wuhan, China; GB115712, 1:500), anti-TSG101 (Servicebio, GB11618, 1:500), and anti-ALIX (Servicebio, GB124080, 1:500).

### 2.11. RNA Sequencing

Hippocampal tissues were collected from mice treated with EVs (2.0 × 10^8^ particles per 10 μL) for 8 weeks; untreated mice served as controls. Total RNA was extracted using TRIzol reagent (Invitrogen, Carlsbad, CA, USA) according to the manufacturer’s instructions. RNA purity and integrity were assessed using a NanoDrop spectrophotometer and an Agilent 2100 Bioanalyzer (Agilent Technologies, Beijing, China). Poly(A) mRNA was enriched with oligo(dT)-attached magnetic beads, fragmented, and used for first- and second-strand cDNA synthesis, end repair, adaptor ligation, and library construction. DNA nanoballs (DNBs) were generated by Phi29 amplification and loaded onto a patterned nanoarray. Single-end 50 bp sequencing was performed on the BGISEQ-500 platform (BGI, Shanghai, China).

### 2.12. Plasma Biomarker Quantification

Whole blood was collected and centrifuged to obtain the plasma. Plasma concentrations of Aβ1–40, Aβ1–42, phosphorylated Tau181 (p-Tau181), and total Tau (T-Tau) were measured using commercial enzyme-linked immunosorbent assay (ELISA) kits (Mouse Aβ1–40, CSB-E08300m, CUSABIO, Wuhan, China; Mouse Aβ1–42, CSB-E10787m, CUSABIO; Mouse p-Tau181, ML106037, MLbio, Shanghai, China; Mouse T-Tau, ML106601, MLbio) according to the manufacturers’ protocols. Samples and standards were added to 96-well plates and incubated at 37 °C for 30 min. After washing, the HRP-conjugated detection reagent was added, followed by substrate development. Reactions were terminated with stop solution, and absorbance was measured at 450 nm using a microplate reader. Concentrations were calculated from standard curves.

### 2.13. Statistical Analysis

Normality was assessed using the Shapiro–Wilk test. Normally distributed data are presented as mean ± standard deviation (SD) and were analyzed using parametric tests. Non-normally distributed data were presented as median with interquartile range (Q1–Q3) and analyzed using nonparametric tests. For two-group comparisons, the unpaired Student t-test or the Mann–Whitney U test was used as appropriate. For comparisons among multiple groups, one-way analysis of variance (ANOVA) followed by Dunnett’s (vs. control) or Tukey’s (all pairwise comparisons) post hoc test was used for normally distributed data, whereas the Kruskal–Wallis test with Dunn’s correction was used for non-normally distributed data. Potential outliers were assessed using the ROUT method (Q = 1%); no data were excluded unless technical failure was documented. For RNA sequencing, differential gene expression was determined using a standard negative-binomial workflow with Benjamini–Hochberg false-discovery-rate control (adjusted *p* < 0.05) and a |log2 fold change| threshold as specified in the Results. General statistical analyses were performed using GraphPad Prism 9 (GraphPad Software, San Diego, CA, USA), and RNA-seq analyses were conducted using an established RNA-seq pipeline. A *p* value < 0.05 was considered statistically significant.

## 3. Results

### 3.1. Characterization of ADMSCs and ADMSC-Derived EVs

**Figure 1:**
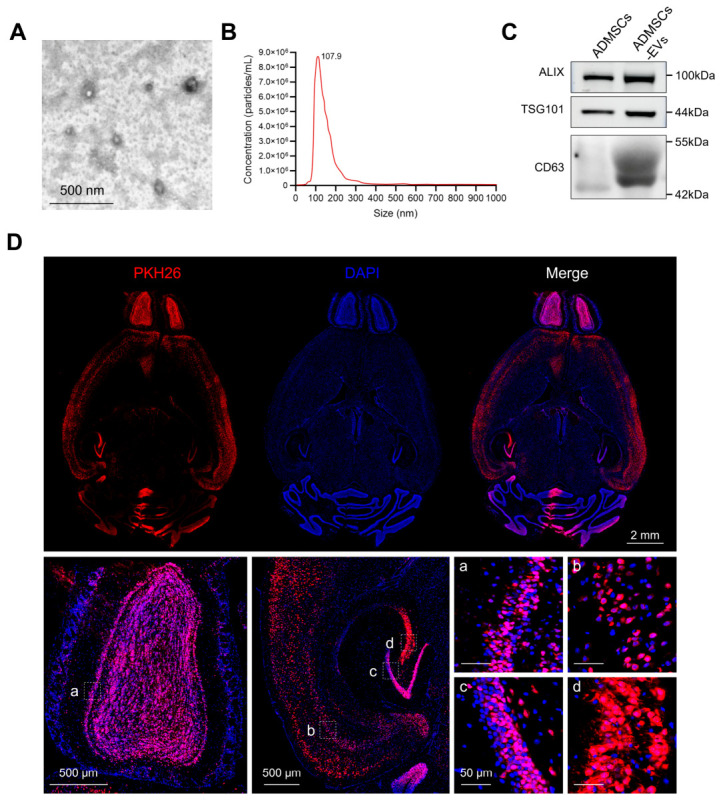
Isolation, validation, and characterization of ADMSC-derived EVs. (**A**) Representative transmission electron microscopy (TEM) image showing round or cup-shaped ADMSC-derived EVs with lipid bilayer structures. Scale bar = 500 nm. (**B**) Nanoparticle tracking analysis (NTA) demonstrating the size distribution and concentration of ADMSC-derived EVs, with a peak diameter of 107.9 nm. (**C**) Western blot analysis of EV markers apoptosis-linked gene 2-interacting protein X (ALIX, ~100 kDa), tumor susceptibility gene 101 (TSG101, ~44 kDa), and cluster of differentiation 63 (CD63, ~40–55 kDa) in ADMSCs and corresponding EV preparations. (**D**) Representative coronal brain sections collected after intranasal administration of PKH26-labeled EVs. PKH26 (red), DAPI (blue), and merged images are shown (top). Enlarged regions (**a**–**d**) highlight EV distribution in olfactory and hippocampal regions. Scale bars: whole brain, 2 mm; regional views, 500 µm; insets, 50 µm. Original images of (**C**) can be found in Supplementary Materials.

Primary ADMSCs exhibited typical fibroblast-like morphology, forming homogeneous monolayers of elongated, spindle-shaped cells arranged in parallel (Figure S1A). Trilineage induction assays confirmed the mesenchymal identity and multilineage differentiation potential. Adipogenic differentiation resulted in abundant intracellular lipid droplets, as detected by Oil Red O staining. Chondrogenic induction resulted in robust extracellular matrix deposition, as shown by Alcian blue staining, whereas osteogenic differentiation led to extensive mineralized nodule formation, as visualized by Alizarin Red S staining (Figure S1B). Flow cytometry demonstrated high expression of the mesenchymal markers CD105 and CD90 and minimal expression of CD11b and CD19 (97.92% of cells were CD105^+^/CD11b^−^ and 98.72% were CD90^+^/CD19^−^), fulfilling the established criteria for ADMSC identification (Figure S1C). EVs isolated from passage 5 ADMSCs exhibited characteristic round or cup-shaped morphology under TEM (Figure 1A). NTA revealed a unimodal size distribution with a peak diameter of 107.9 nm and a mean size of 113.8 ± 33.7 nm, consistent with the small EVs. The particle concentration was approximately 1.3 × 10^11^ particles/mL (Figure 1B). Western blot analysis confirmed enrichment of the canonical exosomal markers ALIX, TSG101, and CD63 in EV preparations compared with parental ADMSCs (Figure 1C). Following intranasal administration, strong PKH26 fluorescence signals were observed along the olfactory pathway and in multiple brain regions, including the olfactory bulb, piriform cortex, and hippocampus (Figure 1D), indicating efficient nose-to-brain transport and widespread parenchymal distribution. Together, these findings establish the morphological, molecular, and biodistribution characteristics of ADMSC-derived EVs and support their suitability for intranasal brain delivery.

### 3.2. Cellular Distribution of PKH26-Labeled EVs in the Hippocampus of APP/PS1 Mice

**Figure 2:**
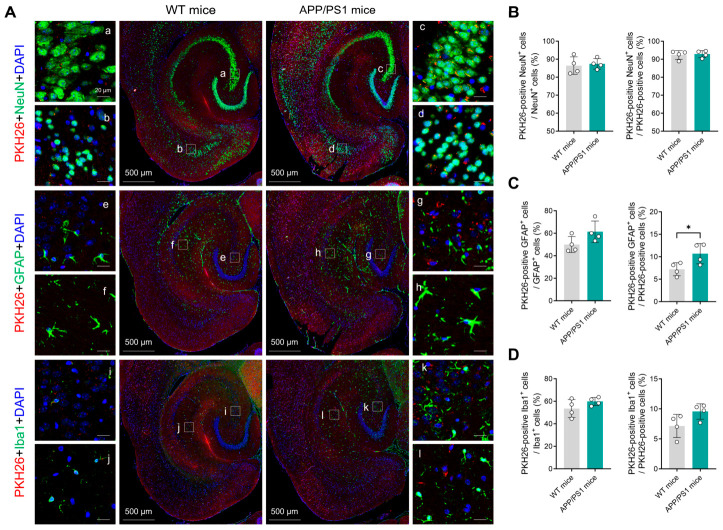
Cellular uptake of PKH26-labeled EVs in the hippocampus of WT and APP/PS1 mice. (**A**) Representative immunofluorescence images of hippocampal sections (CA1, CA3, and dentate gyrus) from WT and APP/PS1 mice showing co-localization of PKH26-labeled EVs (red) with NeuN-positive neurons, GFAP-positive astrocytes, or Iba1-positive microglia (green). Nuclei were counterstained with DAPI (blue). (**a**–**l**) indicate enlarged views of the boxed areas shown in the corresponding overview images. Scale bars, 500 μm (low magnification) and 20 μm (high magnification). (**B**) Quantification of the proportion of NeuN-positive neurons that internalized EVs (left) and the percentage of NeuN-positive neurons among PKH26-positive cells (right). (**C**) Quantification of the proportion of GFAP-positive astrocytes that internalized EVs (left) and the percentage of astrocytes among PKH26-positive cells (right). (**D**) Quantification of the proportion of Iba1-positive microglia that internalized EVs (left) and the percentage of microglia among PKH26-positive cells (right). Data are presented as mean ± SD; *n* = 4; * *p*  <  0.05.

To characterize the cellular uptake of intranasally delivered EVs, hippocampal sections from WT and APP/PS1 mice were analyzed by immunofluorescence co-staining. As shown in Figure 2A, PKH26 fluorescence was detected within NeuN-positive neurons throughout the hippocampal formation, including the CA1, CA3, and dentate gyrus regions, in both WT and APP/PS1 mice, indicating the neuronal internalization of administered EVs. Quantitative analysis revealed that approximately 90% of NeuN-positive neurons exhibited EV uptake in both WT and APP/PS1 mice (Figure 2B, left). Conversely, nearly 90% of PKH26-positive cells were NeuN-positive neurons (Figure 2B, right), indicating that neurons represent the predominant cell type that internalizes EVs in the hippocampus. No significant differences were observed between the WT and APP/PS1 mice, suggesting that AD-associated pathology did not markedly alter neuronal EV uptake. We next examined EV uptake by glial cells. PKH26 signals co-localized with GFAP-positive astrocytes and Iba1-positive microglia in both genotypes (Figure 2A). Approximately half of the astrocytes and microglia exhibited detectable EV uptake, with no significant differences between the WT and APP/PS1 mice (Figure 2C,D, left). However, the analysis of the cellular composition of PKH26-positive cells reveals that astrocytes and microglia accounted for less than 10% of the EV-containing cells in WT mice (Figure 2C,D, right). In contrast, APP/PS1 mice showed a greater relative contribution of glial cells to the EV-positive population, particularly astrocytes (Figure 2C, right). Collectively, intranasally delivered EVs were broadly internalized by neuronal and glial populations in the hippocampus, with neurons representing the primary cellular target in both WT and APP/PS1 mice.

### 3.3. ADMSC-EVs Improve Cognitive Deficits and Amyloid Pathology in APP/PS1 Mice with a Nonlinear Dose–Response Pattern

**Figure 3:**
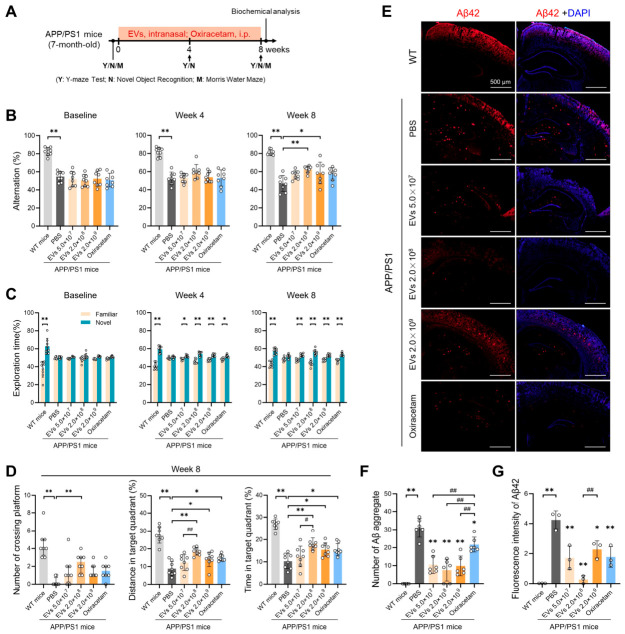
ADMSC-EVs improve cognitive performance and hippocampal Aβ pathology in APP/PS1 mice in a dose-dependent manner. (**A**) Experimental timeline. Seven-month-old APP/PS1 mice received intranasal administration of ADMSC-derived EVs (5.0 × 10^7^, 2.0 × 10^8^, or 2.0 × 10^9^ particles/10 μL), PBS, or oxiracetam (100 mg/kg, intraperitoneally). Behavioral tests were performed at baseline and at 4 and 8 weeks post-treatment. (**B**) Alternation percentage in the Y-maze spontaneous alternation test was measured as an index of spatial working memory at baseline, week 4, and week 8. (**C**) Discrimination performance in the novel object recognition test was evaluated at the indicated time points. (**D**) Number of platform crossings, distance traveled and time spent in the target quadrant during the Morris water maze test trial at week 8. *n* = 8. (**E**) Representative immunofluorescence images of hippocampal sections stained for Aβ42 (red) and nuclei (DAPI, blue) from WT mice and APP/PS1 mice treated with PBS, EVs (5.0 × 10^7^, 2.0 × 10^8^, and 2.0 × 10^9^ particles/10 μL), or oxiracetam. Scale bars = 500 μm. (**F**) Quantification of Aβ42 aggregates number in the hippocampus based on Aβ42 immunofluorescence staining. (**G**) Quantification of hippocampal Aβ42 fluorescence intensity. Data are presented as mean ± SD. *n* ≥ 3, * *p* < 0.05, ** *p* < 0.01 versus APP/PS1 + PBS group; # *p* < 0.05, ## *p* < 0.01 between indicated groups.

APP/PS1 mice exhibit progressive impairments in memory and spatial learning compared to WT controls. To determine therapeutic efficacy, 7-month-old APP/PS1 mice received intranasal EVs at three doses (5.0 × 10^7^, 2.0 × 10^8^, and 2.0 × 10^9^ particles/10 μL) or oxiracetam as a positive control. Behavioral performance was assessed at baseline and after 4 and 8 weeks of treatment (Figure 3A). Baseline testing confirmed comparable impairments across the APP/PS1 groups before the intervention. In the Y-maze, EV-treated APP/PS1 mice showed increased spontaneous alternation at week 8, indicating improved spatial working memory (Figure 3B). In the NORT, discrimination performance progressively improved in EV-treated APP/PS1 mice, with greater enhancement observed at week 8 compared with baseline and week 4 (Figure 3C). Left–right object exploration was comparable among all groups, excluding the effects of exploratory bias (Figure S2A). Spatial reference memory was evaluated using the MWM test. Baseline performance confirmed comparable deficits among the APP/PS1 groups before treatment (Figure S2B). EV treatment increased platform crossings and time and distance spent in the target quadrant relative to PBS-treated APP/PS1 mice (Figure 3D). A nonlinear dose–response relationship was observed: the intermediate dose (2 × 10^8^ particles/10 μL) produced the greatest behavioral benefit, outperforming both the low and high doses and showing effects within the range observed for the oxiracetam reference group across multiple measures.

To evaluate whether EVs alleviate amyloid pathology in APP/PS1 mice, hippocampal Aβ deposition was first examined by immunofluorescence staining. PBS-treated APP/PS1 mice exhibited abundant Aβ42-positive plaques compared with WT controls, whereas EV treatment reduced plaque burden and Aβ42 immunoreactivity across all doses (Figure 3E). Quantification confirmed significant reductions in both the number of Aβ aggregates and Aβ42 fluorescence intensity relative to PBS-treated APP/PS1 mice, with the intermediate dose producing the strongest effect (Figure 3F,G). Congo red staining demonstrated extensive congophilic deposits in the CA1–CA3 and dentate gyrus regions of PBS-treated APP/PS1 mice, which were markedly attenuated by EV treatment, with maximal efficacy at the intermediate dose (Figure S3). Plasma biomarker analysis further demonstrated decreased Aβ42 levels, reduced Aβ42/Aβ40 ratio, lower total Tau, and reduced phosphorylated Tau181 levels in EV-treated mice (Figure S4). The plasma p-Tau181/total Tau ratio was also decreased, showing a reduction similar to that observed in oxiracetam-treated mice. Collectively, these findings demonstrate that intranasal ADMSC-EVs alleviate cognitive deficits and attenuate amyloid-related pathology in APP/PS1 mice, with maximal efficacy at the intermediate dose.

### 3.4. ADMSC-EVs Attenuate Astrocytic and Microglial Activation in the Hippocampus

**Figure 4:**
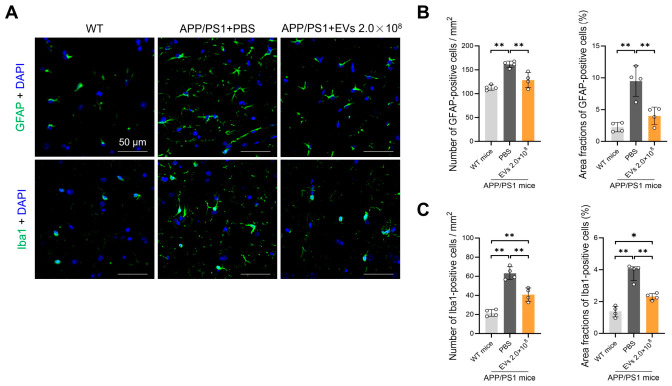
ADMSC EVs reduce astrocytic and microglial activation in the hippocampus of APP/PS1 mice. (**A**) Representative immunofluorescence images of hippocampal sections stained for GFAP (astrocytes, green) or Iba1 (microglia, green), with nuclei counterstained with DAPI (blue), from WT mice, PBS-treated APP/PS1 mice, and APP/PS1 mice treated with ADMSC-derived EVs (2.0 × 10^8^ particles/10 μL). Scale bar = 50 μm. (**B**) Quantification of GFAP-positive astrocytes, including cell density (cells/mm^2^) and area fraction (%). (**C**) Quantification of Iba1-positive microglia, including cell density (cells/mm^2^) and area fraction (%). Data are presented as mean ± SD. *n* = 4; * *p* < 0.05, ** *p* < 0.01.

Reactive gliosis and microglial activation are prominent neuropathological features of AD. Immunofluorescence analysis of hippocampal sections revealed marked astrocyte and microglial activation in PBS-treated APP/PS1 mice, as evidenced by increased numbers and hypertrophic morphology of GFAP-positive astrocytes and Iba1-positive microglia compared with WT mice (Figure 4A). In contrast, EV-treated APP/PS1 mice (2.0 × 10^8^ particles/10 μL) exhibited reduced glial activation. Quantitative analysis showed significant decreases in both the density (cells/mm^2^) and area fraction of GFAP-positive astrocytes relative to PBS-treated APP/PS1 mice (Figure 4B). Similarly, EV treatment significantly reduced the number and area fraction of Iba1-positive microglia (Figure 4C). These parameters were partially restored toward WT levels, indicating that intranasal ADMSC-EVs attenuate hippocampal neuroinflammatory responses associated with AD pathology.

### 3.5. ADMSC-EVs Confer Sustained Cognitive and Neuropathological Benefits After Treatment Withdrawal

**Figure 5:**
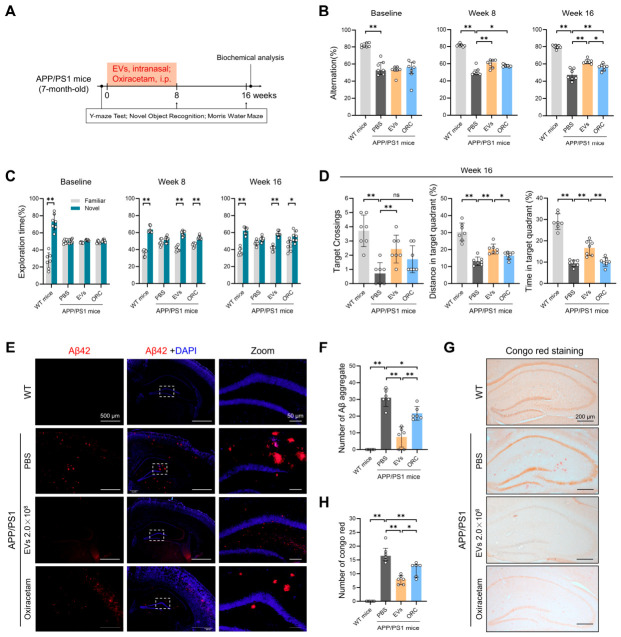
Long-term behavioral performance and amyloid and Tau pathology following cessation of ADMSC-EV treatment. (**A**) Experimental timeline. APP/PS1 mice received intranasal ADMSC-derived EVs (2.0 × 10^8^ particles/10 μL) or intraperitoneal oxiracetam for 8 consecutive weeks, followed by an 8-week treatment-free observation period. Behavioral tests were performed at baseline, at the end of treatment (week 8), and 8 weeks after treatment withdrawal (week 16). (**B**) Y-maze spontaneous alternation performance at baseline, week 8, and week 16. (**C**) Novel object recognition test showing exploration time for familiar and novel objects at each time point. (**D**) Morris water maze probe trial metrics, including target-platform crossings, distance in the target quadrant, and time in the target quadrant, at week 16. *n* = 7. (**E**) Representative hippocampal immunofluorescence images showing Aβ42 (red) and nuclei (DAPI, blue). Dashed boxes indicate the regions shown at higher magnification. (**F**) Quantification of hippocampal Aβ aggregate number. (**G**) Representative Congo red staining of the hippocampal sections. (**H**) Quantification of Congo red-positive amyloid plaques. Data are presented as mean ± SD. *n* ≥ 3, * *p* < 0.05, ** *p* < 0.01, ns not significant.

To assess the durability of EV-mediated cognitive improvement, APP/PS1 mice received intranasal EVs (2.0 × 10^8^ particles/10 μL) or oxiracetam for 8 weeks, followed by an 8-week treatment-free period (Figure 5A). Behavioral performance was evaluated at baseline, at week 8 (end of treatment), and at week 16 (8 weeks after withdrawal). In the Y-maze, both EV and oxiracetam treatment improved alternation performance at week 8. Importantly, EV-treated mice maintained elevated alternation rates at week 16, whereas the effect of oxiracetam was partially attenuated, although performance remained higher than that of PBS-treated APP/PS1 mice (Figure 5B). Similarly, in the novel object recognition test, both treatments enhanced novel object exploration at week 8. At week 16, recognition memory was better preserved in the EV-treated group, whereas oxiracetam-treated mice exhibited weaker and less consistent retention (Figure 5C). In the MWM probe trial, EV-treated mice retained increased platform crossings and greater time and distance in the target quadrant relative to PBS controls at both week 8 (Figure S5C) and week 16 (Figure 5D). In contrast, oxiracetam-treated mice demonstrated partial loss of efficacy, with some parameters no longer significantly different from PBS controls at week 16. These findings indicate that a finite treatment course of EV administration induces sustained cognitive benefits that persist beyond the treatment period.

To determine whether neuropathological changes persisted after treatment cessation, hippocampal tissue was analyzed at week 16, 8 weeks following EVs or oxiracetam withdrawal. Immunofluorescence analysis revealed extensive Aβ42 accumulation in PBS-treated APP/PS1 mice, whereas EV-treated mice exhibited a sustained reduction in Aβ42-positive aggregates (Figure 5E). Quantitative analysis confirmed significantly fewer Aβ deposits in EV-treated mice compared with PBS controls (Figure 5F). Consistently, Congo red staining demonstrated reduced fibrillar plaque burden across hippocampal subregions following EV treatment, persisting after the treatment-free interval (Figure 5G,H). Together, these findings indicate that EV administration induces persistent attenuation of amyloid deposition that extends beyond the active treatment period.

### 3.6. ADMSC-EV Treatment Is Associated with Neuroregenerative and Inflammatory Pathways

**Figure 6:**
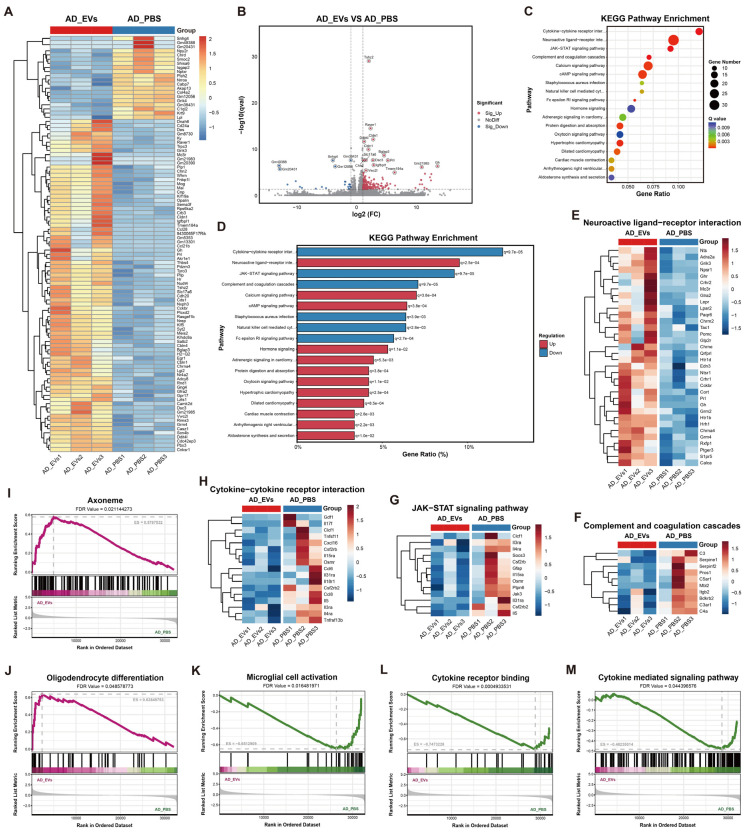
ADMSC-EVs promote neuronal regeneration and suppress inflammation in the hippocampal tissue. (**A**) Heatmap of EXO-induced gene expression alterations. (**B**) Volcano plot of differentially expressed genes (DEGs) modulated by EXOs. (**C**) KEGG enrichment dot plot for EXO-responsive DEGs. (**D**) KEGG enrichment bar plot for EXO-responsive DEGs. (**E**) Heatmap of gene expression in the KEGG “Neuroactive ligand-receptor interaction” pathway. (**F**) Heatmap of gene expression in the KEGG “Complement and coagulation cascades” pathway. (**G**) Heatmap of gene expression in the KEGG “JAK-STAT signaling pathway”. (**H**) Heatmap of gene expression in the KEGG “Cytokine-cytokine receptor interaction” pathway. (**I**) GSEA enrichment plot for the GO term “Axoneme”. (**J**) GSEA enrichment plot for the GO term “Oligodendrocyte differentiation”. (**K**) GSEA enrichment plot for the GO term “Microglial cell activation”. (**L**) GSEA enrichment plot for the GO term “Cytokine receptor binding”. (**M**) GSEA enrichment plot for the GO term “Cytokine-mediated signaling pathway”. Magenta curves indicate gene sets enriched/upregulated in the AD_EVs group, whereas green curves indicate gene sets enriched/upregulated in the AD_PBS group. Dashed lines indicate the peak enrichment score.

RNA sequencing detected 32,905 expressed genes in the hippocampal tissues. Among these, 17,612 genes showed positive log_2_ fold changes, and 15,293 showed negative log_2_ fold changes in EV-treated mice relative to controls. Using a threshold of adjusted q < 0.05 and |fold change| ≥ 2, 198 significantly differentially expressed genes (DEGs) were identified, including 175 upregulated and 23 downregulated genes. A heatmap illustrates the top 100 DEGs ranked by statistical significance (Figure 6A), and a volcano plot highlights the distribution of DEGs, with the top 20 most significant genes (Figure 6B). KEGG pathway analysis revealed significant enrichment in multiple signaling pathways, including the cytokine–cytokine receptor interaction (q = 9.7 × 10^−5^), neuroactive ligand–receptor interaction (q = 2.5 × 10^−4^), JAK-STAT signaling pathway (q = 9.7 × 10^−5^), complement and coagulation cascades (q = 9.7 × 10^−5^), calcium signaling pathway (q = 3.8 × 10^−4^), and cAMP signaling pathway (q = 3.8 × 10^−4^) (Figure 6C). EV treatment was associated with increased expression of genes involved in neurotransmission and intracellular signaling, including neuroactive ligand–receptor interaction, calcium signaling, and cAMP signaling, whereas genes associated with inflammatory pathways, such as cytokine–cytokine receptor interaction, JAK–STAT signaling, and complement cascades, were relatively downregulated (Figure 6D). The expression patterns within these pathways are shown in Figure 6E–H. Additionally, GSEA enrichment analysis further identified significant associations with gene ontology (GO) terms related to axoneme assembly (FDR = 0.021; Figure 6I), oligodendrocyte differentiation (FDR = 0.049; Figure 6J), microglial cell activation (FDR = 0.016; Figure 6K), cytokine receptor binding (FDR = 4.9 × 10^−4^; Figure 6L), and cytokine-mediated signaling pathways (FDR = 0.044; Figure 6M). Together, these transcriptomic findings reveal pathway changes associated with EV treatment, including neural regeneration and inflammation-related features, providing directions for future mechanistic exploration.

## 4. Discussion

In this study, intranasally delivered ADMSC-EVs accessed multiple brain regions through the nose-to-brain route, produced nonlinear, dose-dependent improvements across three cognitive paradigms, and attenuated amyloid pathology in APP/PS1 mice. An intermediate dose (2.0 × 10^8^ particles/10 μL) consistently yielded the largest gains, whereas a higher dose did not confer any additional benefit. Central changes were mirrored peripherally by reductions in plasma Aβ and p-Tau181/total Tau ratio, supporting their potential value as minimally invasive pharmacodynamic readouts. This study not only supports the therapeutic potential of ADMSC-EVs for AD but also systematically evaluates their intranasal administration route, dosage optimization, and durability of efficacy, thereby providing useful evidence for the translational development of EV-based interventions.

PKH26 tracing demonstrated sequential distribution in the olfactory bulb, piriform cortex, hippocampus, and parietal–frontal cortex, consistent with established olfactory/trigeminal nose-to-brain transport pathways [32]. This delivery route is consistent with the behavioral specificity we observed, including improved recognition memory, spatial working memory, and spatial reference memory. A key observation of the present study was the nonlinear dose–response pattern of intranasal ADMSC-EVs. The low dose produced limited efficacy, the intermediate dose yielded the most consistent benefits, and the high dose did not further enhance therapeutic outcomes. This pattern is consistent with well-documented non-monotonic relationships in biological and endocrine pathways, and complex system-level responses reported for nanoparticle exposures [33,34,35]. Several factors may contribute to this phenomenon, including saturation of nose-to-brain transport, limited recipient cell uptake capacity, EV aggregation or altered biodistribution at high particle concentrations, and dose-dependent activation of clearance or inflammatory responses. These possibilities highlight the importance of defining an effective dosing window, rather than assuming a linear dose–efficacy relationship. Future studies incorporating quantitative biodistribution, cell-type-specific uptake analysis, EV clearance kinetics, and potency assays will help clarify the biological basis of this nonlinear response. Moreover, these benefits persisted for up to 16 weeks after the defined dosing course. Compared with oxiracetam, the optimized EV dose produced behavioral improvements within a comparable efficacy range, supporting the functional relevance of the intervention. Nevertheless, the biological basis of durability, including whether it reflects persistent EV cargo activity, long-lasting remodeling of recipient cells, reduced amyloid burden, or secondary network stabilization, remains to be further explored.

At the pathological level, ADMSC-EVs reduced hippocampal Aβ42 plaque burden and Aβ42 protein levels, suggesting reduced amyloidogenic pathway activity. Concomitantly, p-Tau181 levels declined, and the p-Tau181/total Tau ratio decreased in both the hippocampus and plasma. EV treatment also mitigated astrocytosis and microgliosis, as indicated by reduced GFAP+ and Iba1+ densities and area fractions, suggesting attenuation of the neuroinflammatory tone. The concordance between central changes and plasma biomarkers is noteworthy. Blood p-Tau181 has shown strong clinical utility for diagnosis and disease tracking, and its reduction, together with decreased plasma Aβ, supports the feasibility of non-invasive efficacy monitoring in future translational studies [36].

These transcriptomic data suggest that EV treatment is associated with broad remodeling of hippocampal neural–glial signaling networks. Rather than targeting a single pathogenic pathway, EV treatment appears to be associated with a shift in the hippocampal microenvironment toward a state that favors efficient neuronal communication, structural support, and reduced inflammatory tone. Many of the altered genes, including Adra2a, Grik3, Chrm2, Htr1d, and Grm2, encode receptors or signaling components involved in synaptic transmission and neuronal excitability [37], suggesting that EVs may be associated with remodeling of neuroactive signaling rather than acting on a single neurotransmitter system. Consistent with this interpretation, GSEA revealed the enrichment of pathways related to oligodendrocyte differentiation and axoneme assembly, both of which are important for myelin integrity, axonal conduction, and higher-order cognitive function [38]. Given that oligodendrocyte dysfunction and myelin disruption are increasingly recognized as contributors to cognitive decline in AD [39], these pathway changes provide a plausible structural and functional context for the sustained behavioral improvements observed. However, direct validation of oligodendrocyte maturation, myelin repair, or axonal remodeling will be required in future studies. In parallel, EV treatment was associated with broad anti-inflammatory changes at the transcriptomic level, including downregulation of multiple cytokine- and chemokine-related genes within the cytokine–cytokine receptor interaction pathway and reduced JAK–STAT-related signaling. These changes are consistent with the observed attenuation of microglial activation, a central driver of neuroinflammation in AD [40,41]. Activated microglia and complement signaling have been implicated in aberrant synaptic pruning and synapse loss in AD [42]. In this context, the reduced expression of complement cascade genes, including C3, C3ar1, C4a, and C5ar1, suggests a potential link between EV-associated transcriptomic changes and reduced inflammatory pressure on hippocampal circuits. Given the established role of the cytokine–microglia–complement axis in synaptic vulnerability and cognitive impairment in AD, its attenuation may provide a plausible link between EV-associated transcriptomic remodeling and improved hippocampal function [43]. Collectively, these omics data support a model in which EVs may improve the hippocampal microenvironment through coordinated changes in inflammatory, complement-related, oligodendrocyte-associated, and neuroactive signaling pathways. This interpretation is consistent with behavioral, pathological, and glial activation data but remains to be tested functionally. Whether these pathways are directly regulated by specific EV cargo or arise secondarily from reduced amyloid burden and improved tissue status remains to be determined.

This study has several strengths: multi-modal readouts (behavioral, histological, biochemical, and transcriptomic analyses), direct evidence of nose-to-brain delivery, evaluation of post-withdrawal durability, and convergence of CNS and plasma biomarkers. Several limitations should also be acknowledged. First, this study used a single trans-genic line and predominantly female mice, which may limit its generalizability. Second, PKH26 labeling provides useful distribution information but may permit dye transfer; future studies incorporating genetic reporters, such as CD63-GFP, would help confirm intact vesicle uptake and recipient-cell specificity. Third, EV heterogeneity remains an important issue, and dosing expressed as particle number alone may not fully capture functional potency; standardization by protein content, RNA content, cargo composition, and potency assays would aid in comparability. Finally, the mechanistic inferences in the present study were based primarily on pathway-level analyses. Although RNA-seq identified changes in inflammatory, complement-related, oligodendrocyte-associated, and neuroactive signaling pathways, the active EV cargo, principal recipient cell types, and causal pathways responsible for behavioral rescue remain to be defined. Future studies using cargo-depleted or cargo-enriched EVs, candidate miRNA/protein manipulation, cell-type-specific response analyses, and pathway-directed interventions are required to determine how transcriptomic changes contribute to the therapeutic effects of ADMSC-EVs.

## 5. Conclusions

In summary, intranasal ADMSC-EVs traverse the nose–brain axis and modulate neuronal and glial networks in APP/PS1 mice. By coupling suppression of inflammatory and complement signaling with enhancement of neuronal communication and repair-associated programs, ADMSC-EVs reduce Aβ accumulation and Tau phosphorylation, align with plasma biomarker improvements, and confer sustained cognitive benefits. These findings define a plausible therapeutic window for dosing and provide mechanistic support for the further development of intranasal EV-based therapy for AD.

## Data Availability

All original data supporting the findings of this study are included in the article and Supplementary Materials. Further inquiries can be directed to the corresponding author.

## Supplementary Materials

The following supporting information can be downloaded at https://www.mdpi.com/article/10.3390/biom16060798/s1, Figure S1: Characterization and identification of ADMSCs; Figure S2: Exploratory behavior and baseline spatial memory parameters across treatment groups; Figure S3: Representative Congo red staining of hippocampal subregions in APP/PS1 mice; Figure S4: Plasma Aβ42 and Tau biomarker profiles across treatment groups; Figure S5: Exploratory behavior and spatial memory after EV withdrawal. Original images of Figure 1C can be found in Supplementary Materials.
