# Supplementary material for: Intranasal Adipose-Derived MSC Extracellular Vesicles Confer Sustained Cognitive Improvement and Suppress Alzheimer’s Pathology in APP/PS1 Mice
Source: Biomolecules. 2026 May 28;16(6):798. doi: 10.3390/biom16060798 (PMC13297158; doi:10.3390/biom16060798)
Supplement: Supplementary file 1 [file biomolecules-16-00798-s001.zip › biomolecules-4285613-supplementary.pdf]

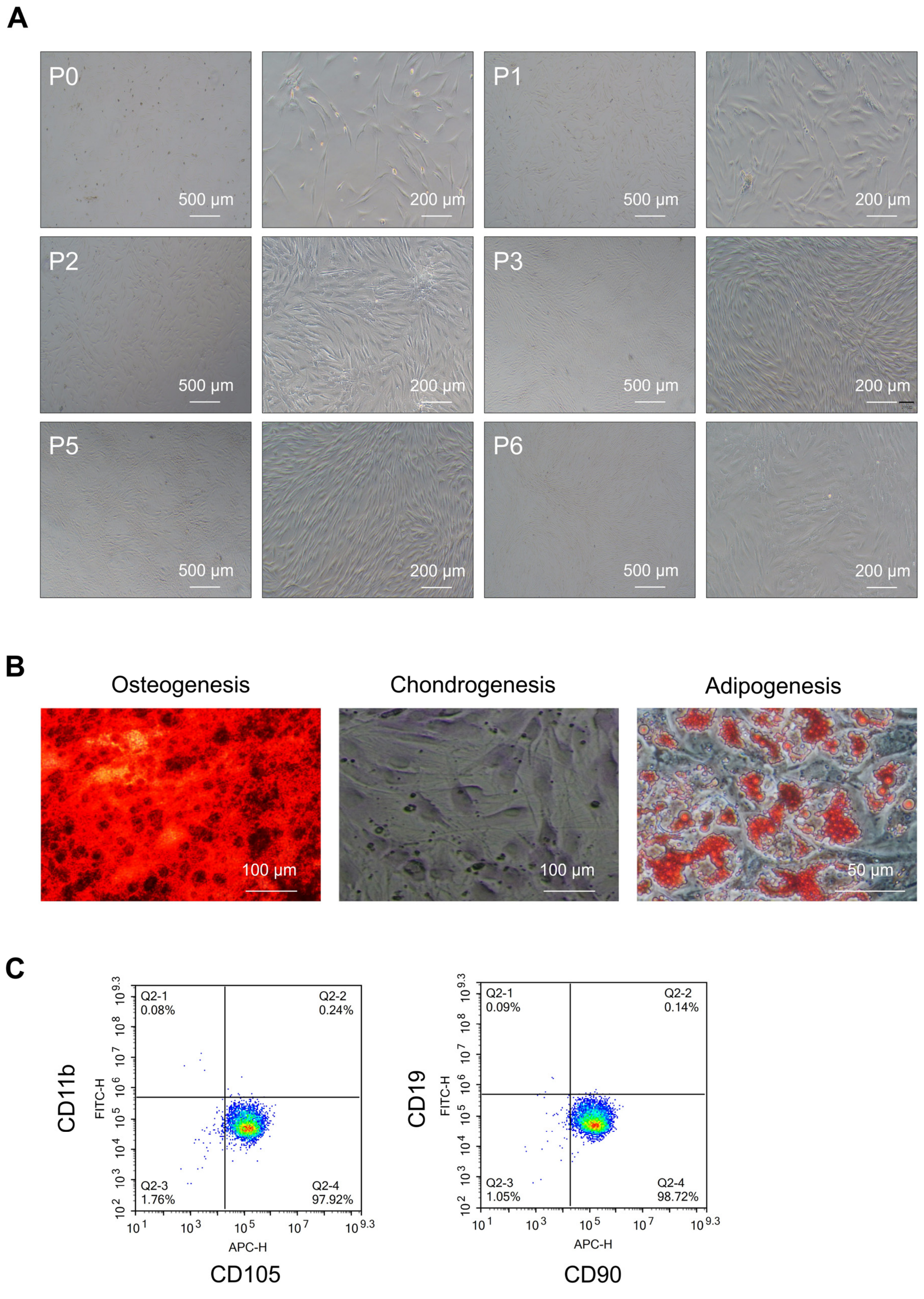

Figure S1. Characterization and identification of ADMSCs. (**A**) Representative phase-contrast images of primary ADMSCs at passages P0–P6, showing typical fibroblast-like, spindle-shaped morphology. Scale bars, 500 μm and 200 μm, as indicated. (**B**) Trilineage differentiation potential of ADMSCs assessed by osteogenic (Alizarin Red S), chondrogenic (Alcian blue), and adipogenic (Oil Red O) staining. (**C**) Flow cytometric analysis of ADMSC surface markers, demonstrating high expression of CD105 and CD90 and negligible expression of CD11b and CD19.

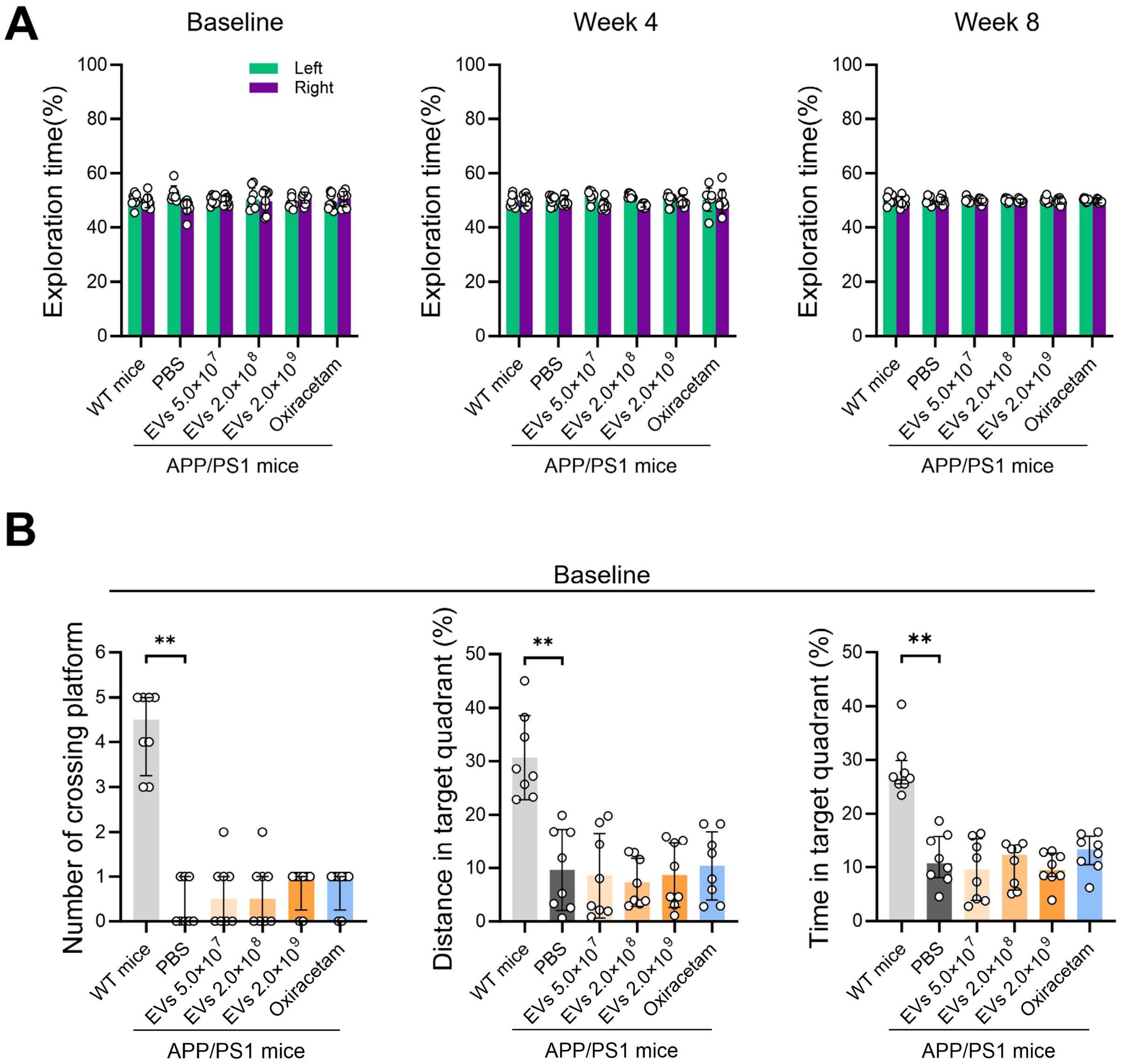

Figure S2. Exploratory behavior and baseline spatial memory parameters across treatment groups. (**A**) Percentage of exploration time spent investigating the identical objects placed in the left and right positions during the exploration phase of the novel object recognition test at baseline, Week 4, and Week 8. WT mice and APP/PS1 mice received PBS, different doses of EVs, or oxiracetam. Exploration time for each object location was recorded prior to the discrimination phase. (**B**) Number of platform crossings, distance traveled and time spent in the target quadrant during the morris water maze test trial at baseline. Data are presented as mean ± SD with individual data points shown. *n* = 8 mice per group.

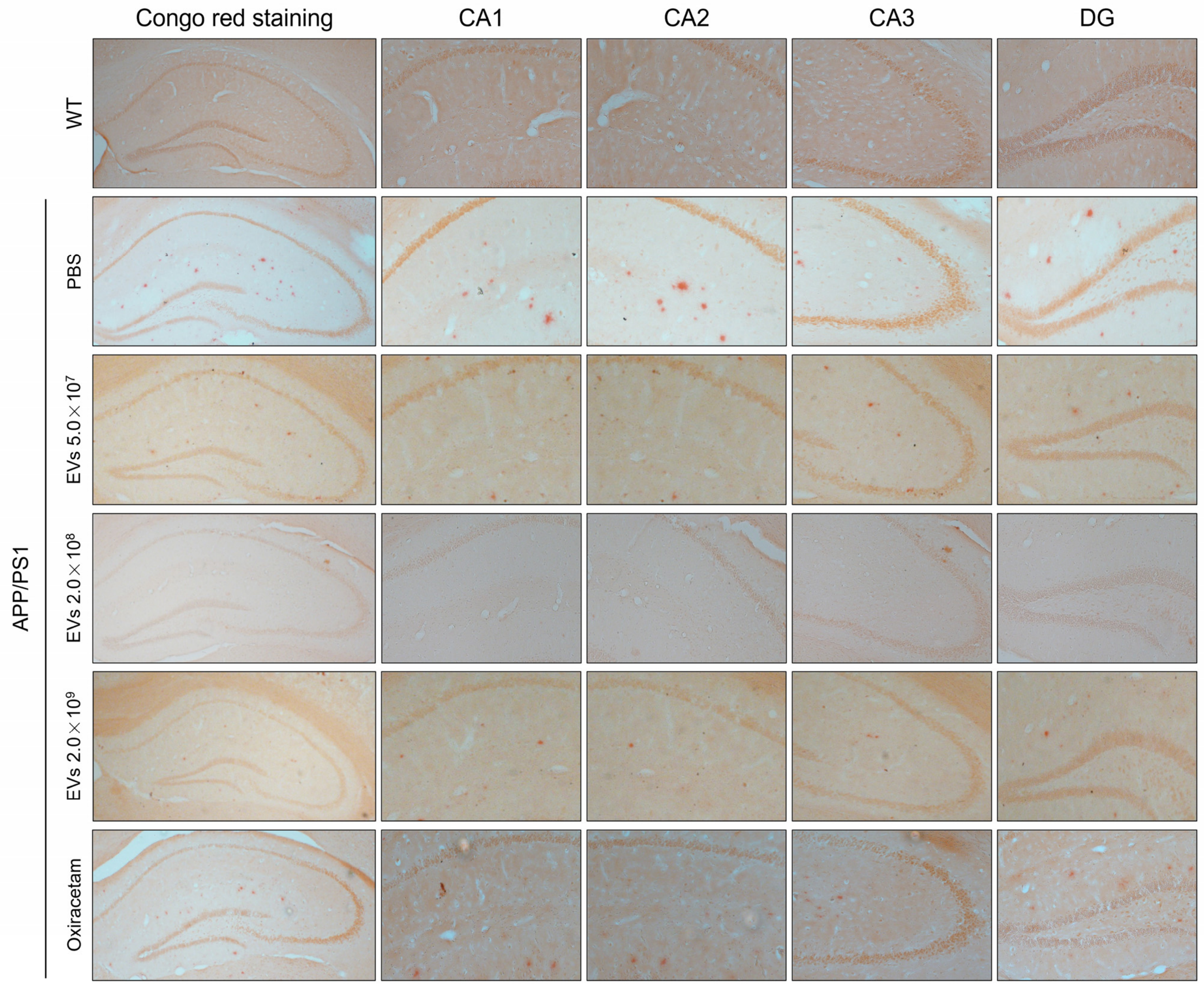

Figure S3. Representative Congo red staining of hippocampal subregions in APP/PS1 mice. Representative images of Congo red staining in hippocampal sections from WT mice and APP/PS1 mice treated with PBS, different doses of EVs (5.0×10^7, 2.0×10^8, and 2.0×10^9 particles/10 μL), or oxiracetam. Low-magnification images illustrate Congo red staining across the entire hippocampus, while higher-magnification images show staining in individual hippocampal subregions, including CA1, CA2, CA3, and dentate gyrus (DG).

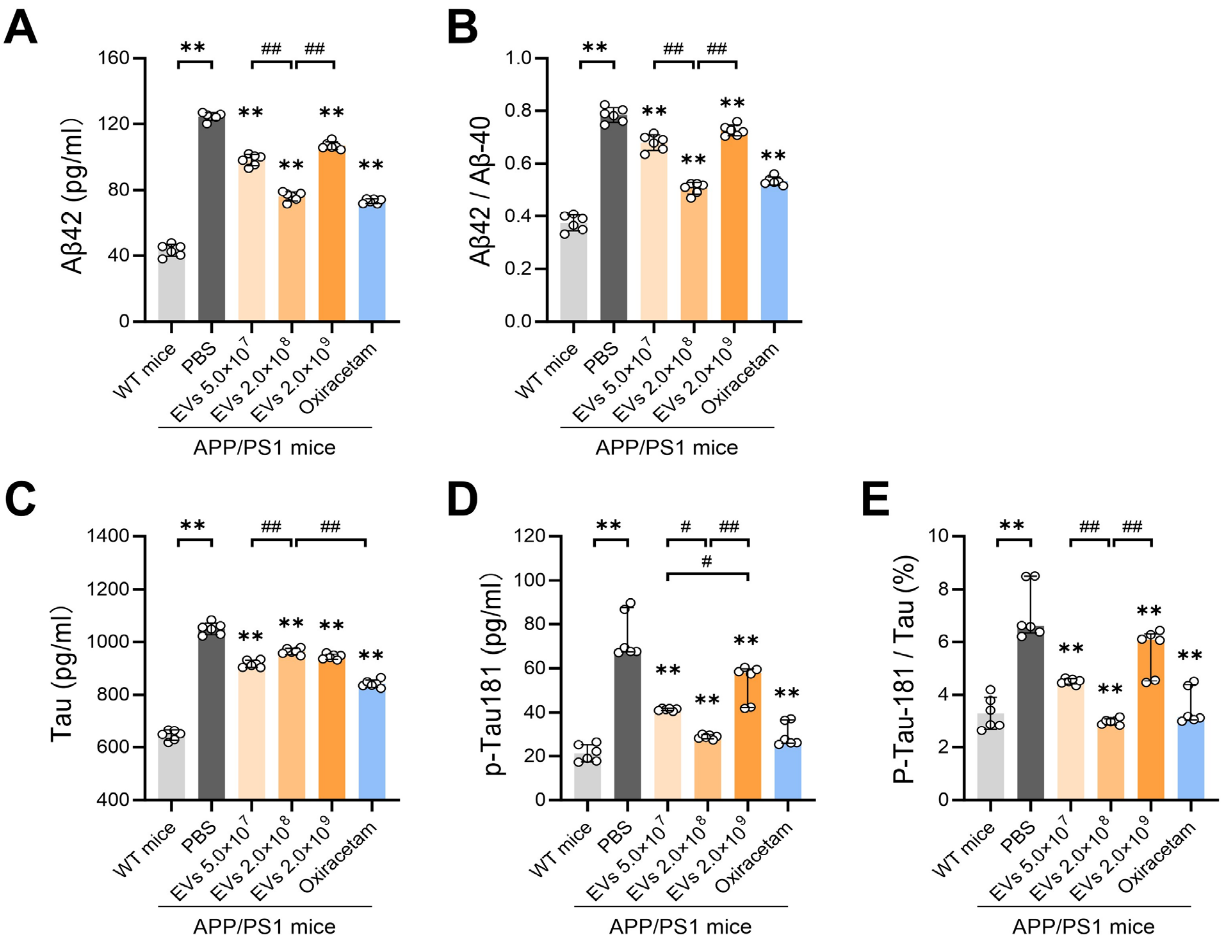

Figure S4. Plasma Aβ42 and Tau biomarker profiles across treatment groups. Plasma biomarkers of Aβ42 (A), Aβ42/Aβ40 ratio (B), total Tau (C), phosphorylated Tau181 (D), and the p-Tau181/total Tau ratio (E) were measured by ELISA in WT mice and APP/PS1 mice treated with PBS, EVs at indicated doses, or oxiracetam. Data are presented as mean ± SD. n =6, **p < 0.01 versus APP/PS1 + PBS group; #p < 0.05, ##P < 0.01 between indicated groups.

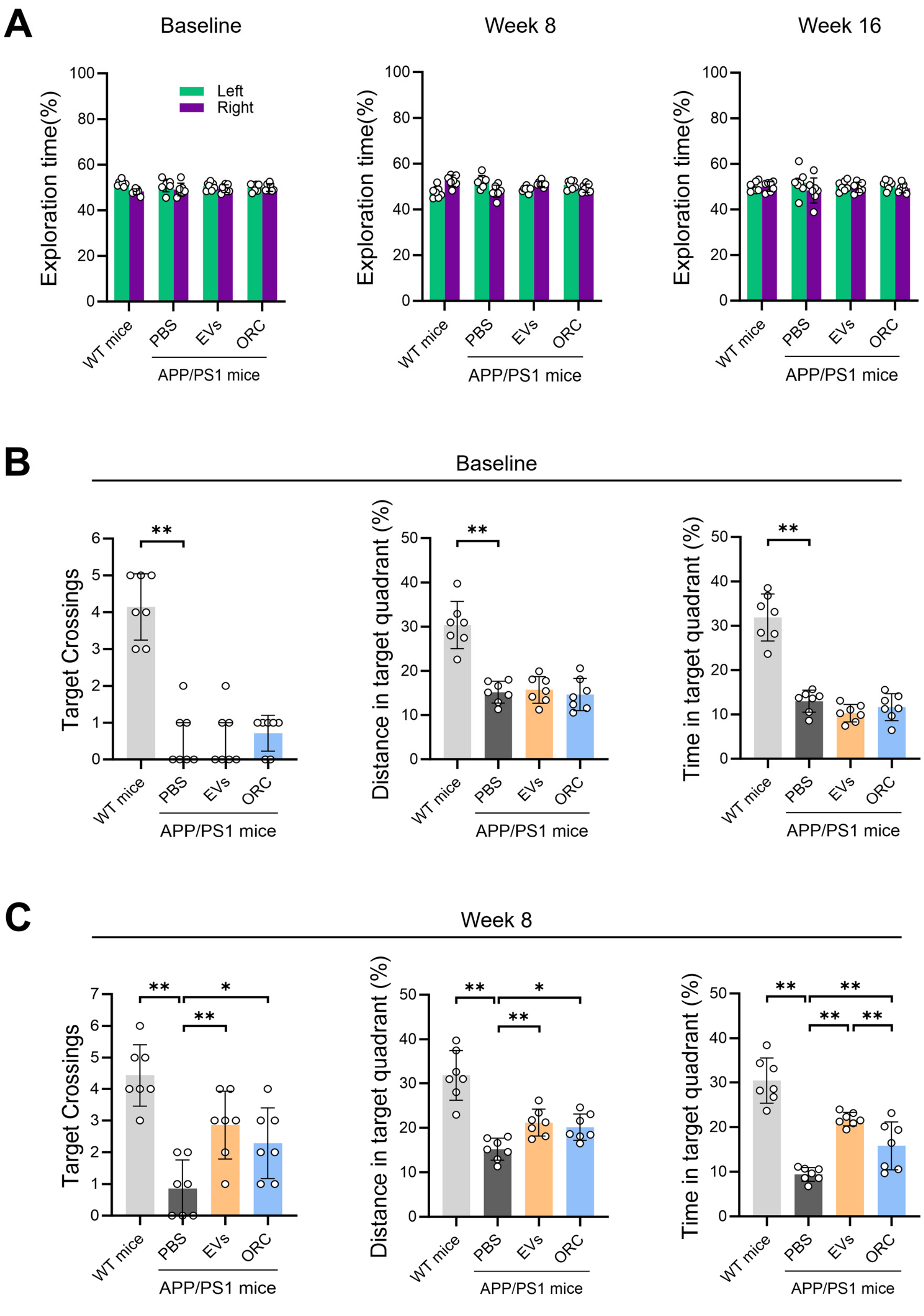

Figure S5. Exploratory behavior and spatial memory after EVs withdrawal. (**A**) Percentage of exploration time spent investigating the identical objects placed in the left and right positions during the exploration phase of the novel object recognition test at baseline, Week 8, and Week 16. WT mice and APP/PS1 mice were treated with PBS, EVs (2.0×10^8 particles/10 μL), or oxiracetam. Exploration time for each object location was quantified at the indicated time points. (**B,C**) Morris water maze probe-trial metrics, including target-platform crossings, distance in the target quadrant, and time in the target quadrant, at baseline (B) and Week 8 (C). Data are presented as mean ± SD with individual data points shown. n = 7 mice per group.

Figure.1C **1**: Marker; **2**: ADMSCs; **3**: ADMSCs-EVs

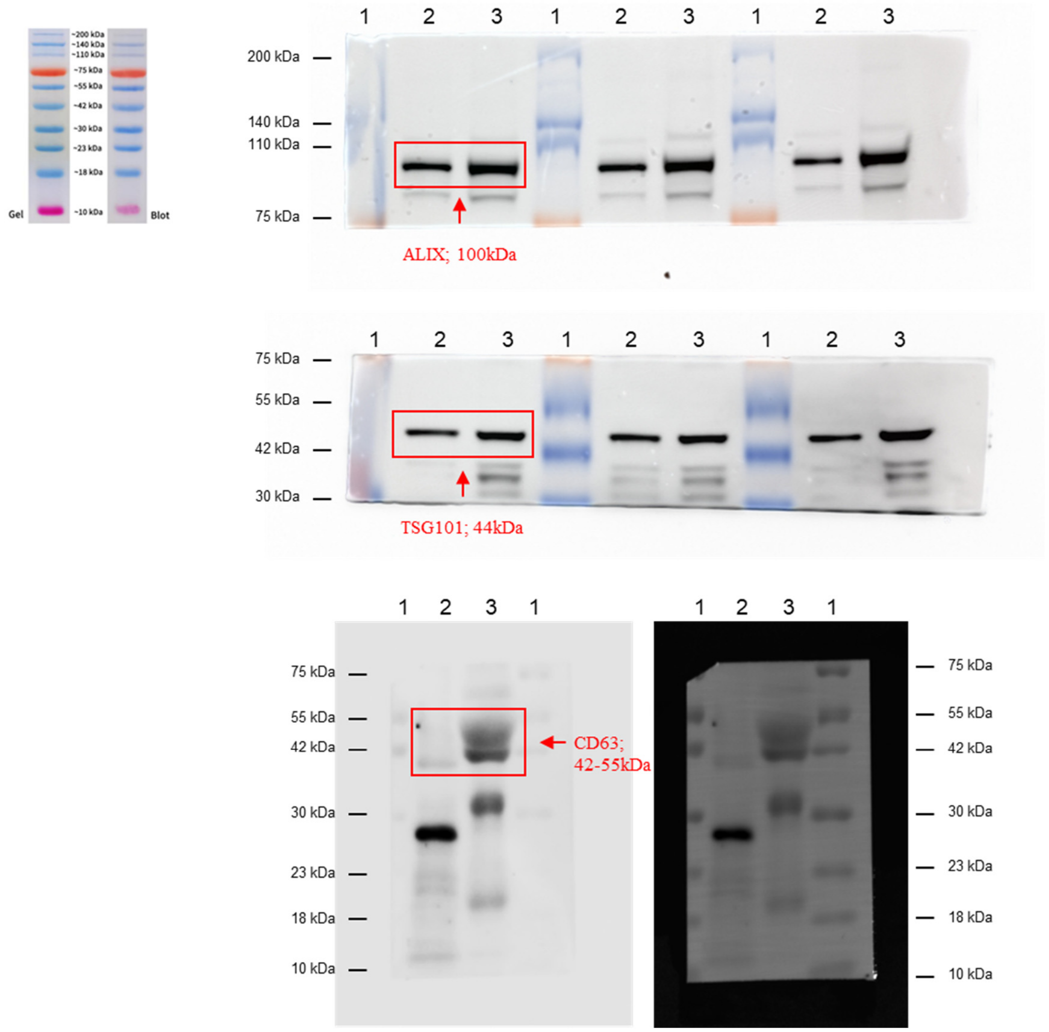

Original western blot images.
